# Comparison of apoptosis in human primary pulmonary endothelial cells and a brain microvascular endothelial cell line co-cultured with *Plasmodium falciparum* field isolates

**DOI:** 10.1186/s12879-017-2552-0

**Published:** 2017-06-27

**Authors:** Jean Claude Biteghe Bi Essone, Nadine N’Dilimabaka, Julien Ondzaga, Jean Bernard Lekana-Douki, Dieudonné Nkoghe Mba, Philippe Deloron, Dominique Mazier, Frédrérick Gay, Fousseyni S. Touré Ndouo

**Affiliations:** 1 International Centre for Medical Research of Franceville (CIRMF), BP 769 Franceville, Gabon; 2 Ecole Doctorale Régionale (EDR) en Infectiologie Tropicale, 876 Franceville, BP Gabon; 3 Département de Parasitologie-Mycologie et de Médecine Tropicale, Faculté de Médecine, Université des Sciences de la Santé, B.P, 4009 Libreville, Gabon; 4 IRD, UMR 216–Mère et Enfant Face aux Infections Tropicales, Faculté de Pharmacie, 4 Avenue de l’Observatoire, 75006 Paris, France; 5 Université Pierre et Marie Curie-Paris 6, UMR S945, 75003 Paris, France; 6 Institut National de la Santé et de la Recherche Médicale, U945, F-75013 Paris, France; 7 AP-HP Groupe hospitalier Pitié-Salpêtrière, Service Parasitologie-Mycologie, 91, boulevard de l’Hopital, 75634 Paris Cedex 13, France

**Keywords:** Malaria, *Plasmodium falciparum*, Endothelial cell death

## Abstract

**Background:**

*Plasmodium falciparum* infection can progress unpredictably to severe forms including respiratory distress and cerebral malaria. The mechanisms underlying the variable natural course of malaria remain elusive.

**Methods:**

The cerebral microvascular endothelial cells-D3 and lung endothelial cells both from human were cultured separately and challenged with *P. falciparum* field isolates taken directly from malaria patients or 3D7 strain (in vitro maintained culture). The capacity of these *P. falciparum* isolates to induce endothelial cell apoptosis via cytoadherence or not was then assessed.

**Results:**

Overall, 27 *P. falciparum* isolates were collected from patients with uncomplicated malaria (*n* = 25) or severe malaria (*n* = 2). About half the isolates (*n* = 17) were able to bind brain endothelial cells (12 isolates, 44%) or lung endothelial cells (17 isolates, 63%) or both (12 isolates, 44%). Sixteen (59%) of the 27 isolates were apoptogenic for brain and/or lung endothelial cells. The apoptosis stimulus could be cytoadherence, direct cell-cell contact without cytoadherence, or diffusible soluble factors. While some of the apoptogenic isolates used two stimuli (direct contact with or without cytoadherence, plus soluble factors) to induce apoptosis, others used only one. Among the 16 apoptogenic isolates, eight specifically targeted brain endothelial cells, one lung endothelial cells, and seven both.

**Conclusion:**

These results indicate that the brain microvascular cell line was more susceptible to apoptosis triggered by *P. falciparum* than the primary pulmonary endothelial cells and may have relevance to host-parasite interaction.

## Background

*Plasmodium falciparum* malaria is a life-threatening parasitic disease that kills some half a million people each year, mostly children living in sub-Saharan Africa. The outcome of the infection, which may be asymptomatic, symptomatic, or life-threatening, is known to be influenced by parasite, host, environmental and socioeconomic factors [1]. In Gabon, respiratory distress and cerebral malaria represent, respectively 31% and 24% of severe *falciparum* in children [2]. However, the molecular mechanisms underlying this variable pathogenicity are unclear. A combination of the parasitized red blood cell (pRBC) binding (cytoadhesion, agglutination and rosetting), the host environmental and inflammatory response, endothelial cell (EC) activation, and altered hemostasis can lead to blood pulmonary/brain barrier impairment [3]. Cerebral malaria accounts for a significant proportion of malaria mortality and is associated with sequestration of pRBC in brain microvessels, especially pRBC expressing PfEMP-1 domain cassettes 8 and 13 [4, 5]. It has been also demonstrated that pRBC-EC adherence induces caspase 8, 9 and 3 activation, EC apoptosis, and modulates EC expression of TNF-α superfamily genes (Fas, Fas L, DR-6) and apoptosis-related genes (Bad, Bax, Caspase-3, SARP 2, DFF45/ICAD, IFN-γ Receptor 2, Bcl-w, Bik and iNOS) [6]. pRBC-EC adherence also leads to microvessels blockage, hypoxia, proinflammatory cytokine secretion [6–9], EC junction modifications, and EC barrier permeabilization [8, 10, 11].

pRBC-triggered EC apoptosis in the brain, lungs and kidneys has been observed in patients with fatal malaria [12, 13]. Using cocultured human lung EC (HLEC) and pRBC field isolates from Gabon, we showed that although almost all isolates provoked cytoadherence, only a few triggered apoptosis (via cytoadherence or diffusible factors), and that these isolates tended to have been drawn from patients with neurological disorders [9]. The potential of pRBC to trigger HLEC apoptosis varies according to the isolate, and is associated to the expression of *Plasmodium* apoptosis-linked pathogenicity factors (PALPFs) [9, 14, 15]. Human endothelial cells in different tissues may not all express the same surface receptors [16], and this could explain some functional differences. For example, CD36 is expressed by HLEC [17], but not by human cerebral microvascular endothelial cells-D3 (hCMEC/D3) (N’dilimabaka et al. unpublished data). Parasite ligands that specifically recognize CD36 are able to stimulate HLEC. ICAM-1 is expressed on the surface of both HLEC and hCMEC/D3 [17, 18], and *P. falciparum* isolates that express surface ligands specifically recognizing ICAM-1 stimulate both cell types*. P. falciparum/*EC cocultures are widely used to study the pathophysiology of malaria [6, 8, 9, 11, 15, 19–21]. Several types of EC have been used in these models, including lung [17], brain EC [18, 21]. These coculture models can be used: a) to study parasite sequestration (cytoadhesion, agglutination, rosetting, etc.), b) to detect damage (apoptosis) caused to EC, c) to categorize and quantify pRBC-EC interactions (cytoadhesion with or without apoptosis), and d) to study the molecules involved in these interactions. We postulated that different EC types might interact differently with pRBC.

Recently, it was shown that pRBC-induced apoptosis of lung and cerebral EC varies with the in vitro-adapted *P. falciparum* line [21]. Selected parasite characteristics are modified after adaptation to in vitro culture, and the behavior of field isolates in the same conditions must be determined. We performed ex vivo coculture experiments with *P. falciparum*-pRBC isolated from malaria patients, and assessed the capacity of the parasites to cytoadhere and to induce apoptosis of both HLEC and hCMEC/D3.

## Materials and methods

### Patients and sampling

**Fig. 1:**
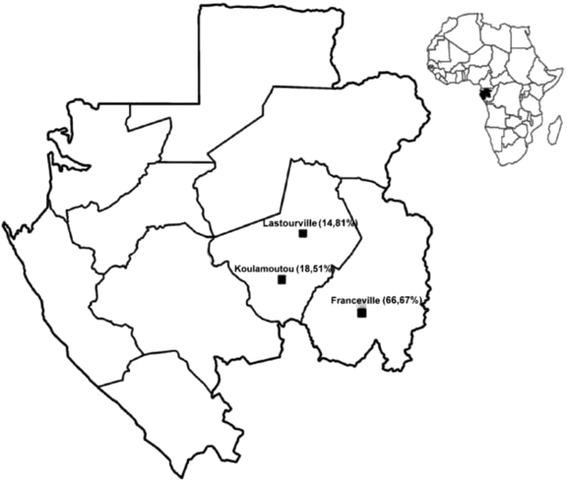
Lastourville (Rural), Koulamoutou (semiurban) and Franceville (urban) location in Gabon. *Percentages in parentheses* indicate the percentage of isolates test by country. Inset shows location of Gabon on the Atlantic Coast of Africa

Patients of all ages were enrolled in three Gabonese regional hospitals: Franceville, Koulamoutou and Lastourville between October 2014 and February 2015 (Fig. 1). Blood samples were collected in EDTA tubes and malaria diagnosis was based on microscopic examination of thick and thin smears, and the clinical status of each patient was determined according to the WHO classification [22].

### *Plasmodium falciparum* maturation

Parasitized red blood cells were selected for their viability, because pRBC from patients having self-medicated (and who rarely inform their physician) do not grow well in culture. Samples with parasite loads of at least 5000 parasites per microliter were washed 4 to 5 times in RPMI 1640 medium to remove white cells. pRBC were immediately grown at 37 °C for 24 to 48 h (until parasites matured into schizonts) in RPMI 1640 with L-glutamine (Sigma) supplemented with 8.3 g/l HEPES, 2.1 g/l sodium bicarbonate, 0.05 g/l hypoxanthine, 0.1 mg/ml gentamicin, 1 mg/ml fungizone, 2 g/l D-glucose and 0.4% Albumax II (InVitrogen, Cergy Pontoise, France), with 5% hematocrit as described [23].

Mature pRBC were enriched by gelatin flotation as previously reported [24] and counted under a microscope. Samples with more than 6% parasitaemia were diluted with non-parasitized red blood cells until to obtain 6% parasitaemia and hematocrit was adjusted at 2%. Also, all the pRBC preparations were free of white blood cell and reticulocytes. All experiments were performed during the first cycle of parasite development and isolates were confirmed to be *Mycoplasma*-free by PCR (data not shown).

### Human endothelial cell culture

#### HLEC culture medium

HLEC were obtained from Professor Frederick Gay (INSERM UMRS 945). These cells predominantly express the CD36 receptor, in addition to other receptors such as Willebrand factor, ICAM1, VCAM1, CD31, E/P-selectin and chondroitin sulfate A [6].

HLEC were cultured in a pulmonary culture medium (PCm) composed of M199 medium (Lonza) supplemented with 10% inactivated FBS (Life Technologies), 5 μg/ml endothelial cell growth supplement (Sigma-Aldrich), 50 U/ml streptomycin-penicillin (Life Technologies), and 0.25 μg/ml fungizone (Lonza). Cells were grown to confluence at 37 °C with 5% CO_2_ and were used at the 10th passage. The cell lines were tested for *Mycoplasma* with the MycoAlert Detection Kit (Lonza) (data not shown).

#### hCMEC/D3 culture medium

The hCMEC/D3 were provided by Professor Pierre Olivier Couraud (INSERM, U1016, Paris, France). This immortalized line of human brain endothelial cells was produced from freshly isolated adult cells. It maintains in stable manner (at least 80 doublings) a differentiated phenotype similar to that of cerebral endothelial cells and forms homogeneous and stable confluent monolayers. hCMEC/D3 express many endothelial markers, including CD31, ICAM1, vWF and VE-cadherin, and blood-brain-barrier (BBB) markers such as proteins associated with tight junctions (ZO-1, JAM-A, claudin-5) [18].

The hCMEC/D3 were cultured in a cerebral culture medium (CCm) composed of EBM-2 basal medium (Lonza) supplemented with 5% inactivated FBS (Life technologies), 1% penicillin-streptomycin (Life Technologies), 0.25 μg/ml fungizone (Lonza), 1.4 μM hydrocortisone (Sigma-Aldrich), 5 μg/ml ascorbic acid (Sigma-Aldrich), 1/100 chemically defined lipid concentrate (Life Technologies), 10 mM HEPES and 1 ng/ml basic fibroblast growth factor (Merck Millipore). The cells were used at the 25th passage. The hCMEC/D3 were grown to confluence at 37 °C with 5% CO_2_ and cell lines were tested for *Mycoplasma* with the MycoAlert Detection Kit (Lonza) (data not shown).

### Cytoadherence assay

Parasitized red blood cells cytoadherence to HLEC and hCMEC/D3 were determined using microscopy as described elsewhere [25]. Briefly, each cell line was grown to confluence (approximately 8.4 × 10^4^ HLEC and 7 × 10^4^ hCMEC/D3) in its respective culture medium in 8-well Lab-Tek™ chamber slides. Confluent HLEC and hCMEC/D3 were then used directly in adhesion assays, or fixed for one hour at 37 °C with 2% paraformaldehyde and kept at 4 °C in 400 μl of phosphate buffered saline (PBS) for a maximum of 2 months [26]. A suspension of 300 μl of mature pRBC at 6% parasitaemia and 2% haematocrit (approximately 4 × 10^6^ schizonts for 63 × 10^6^ uninfected RBC) in complete RPMI culture medium without bicarbonate was added to each well containing confluent HLEC and hCMEC/D3 monolayers from Labtek in duplicate, and incubated for 1 h at 37 °C. Also, cytoadherence was performed using 3D7 strain at the same conditions as positive control.

After 1 h of incubation, unfixed pRBC were removed gently by washing 5 times with PBS and the Labtek was fixed with 2% glutaraldehyde for 20 min at room temperature, rinsed with PBS and stained with 30% Giemsa. The number of adherent pRBCs was determined microscopically, and expressed as the number of pRBC adhering to 700 cells. Cytoadherence was considered positive if the number of adhering pRBC was greater than to 35 per 700 cells (more than 5% of adhering pRBC).

### Apoptosis assay

HLEC and hCMEC/D3 were grown respectively in PCm and CCm until 80–90% confluence in both 24-well (about 3 × 10^5^ cells for HLEC and 2.55 × 10^5^ cells for hCMEC/D3) and 6-well Transwell plates (about 10^6^ cells for HLEC and 9 × 10^5^ cells for hCMEC/D3) with polyester 0.4 μm pore-size filters (Costar, Corning Incorporated). The 24-well plates were used for apoptosis assay in contact condition whereas the 6 well plates (Transwell) were used for apoptosis assay in non-contact experiment.

#### Contact and non-contact co-culture

Co-cultures of *P. falciparum* field isolates with hCMEC/D3 or HLEC were carried out in parallel using two modified media that lacked bicarbonate [11, 21]. Briefly, for hCMEC/D3, the modified medium contained RPMI 1640 + L-glutamine +25 mM HEPES (Gibco) supplemented with 5% inactivated FBS, 1.4 μM hydrocortisone, 5 μg/ml ascorbic acid, 1/100 chemically defined lipid concentrate and 1 ng/ml of basic fibroblast growth factor and was called MCCm. For HLEC, it contained RPMI 1640 + L-glutamine +25 mM HEPES (Gibco) supplemented with 10% of inactivated FBS and 5 μg/ml of endothelial cell growth supplement and was called MPCm.

For contact co-culture experiment, a suspension of 133 μl of mature pRBC (isolates and positive control 3D7 strain) at 6% parasitaemia and 1% haematocrit (approximately 5.31 × 10^5^ schizonts for 9 × 10^6^ uninfected red blood cells (RBC)) in both complete MPCm and complete MCCm has been co-culture respectively with HLEC and hCMEC/D3 separately, in quadruplicate using the 24-well plates.

In non-contact coculture experiment, a suspension of 500 μl of mature pRBC (isolates and positive control 3D7 strain) at 6% parasitaemia and 1% haematocrit (approximately 2 × 10^6^ schizonts for 35 × 10^6^ uninfected RBC) in both complete MPCm and complete MCCm has been coculture respectively with HLEC and hCMEC/D3 separately, in quadruplicate using the 6 well plates Transwell. Each apoptosis assay condition was performed using a suspension of uninfected RBC at 1% haematocrit for negative controls (approximately 9.5 × 10^6^ RBC). Each coculture was then incubated for 24 h at 37 °C with 5% CO2.

#### Apoptosis detection

Apoptosis was measured with an enzyme immunoassay (Cell Death Detection ELISA plus, Roche Diagnostics) as recommended by the manufacturer. This ELISA detects cytoplasmic nucleosomes released from apoptotic cells and has an estimated detection limit of 625 apoptotic cells. Both HLEC and hCMEC cell line was incubated with 3D7 stain as positive control and uninfected RBC as negative controls. After 24 h of incubation, the 24-well plates were washed 5 times with RPMI 1640, and twice with PBS to remove unbound pRBC, 3D7 stain and RBC. In the mean time, the upper compartment of the 6-well Transwell plates containing pRBC, 3D7 stain and RBC was removed, the supernatant was discarded and the cells were also washed twice with PBS. Two hundred (200) μl and 500 μl of lysis buffer provided with the ELISA kit were added to the 24 and 6-well plates respectively to permeabilize HLEC and hCMEC/D3. The plates were then centrifuged at 1000 rpm for 10 min, and 20 μl of supernatant per well was used in the ELISA test. Optical density (OD) was read with a Stat FaxH 3200 (Fisher Bioblock Scientific, Illkirch France) at 405 nm, with a reference filter of 492 nm. The mean OD of pRBC-activated HLEC and hCMEC/D3 to RBC-activated HLEC and hCMEC/D3 and mean OD of 3D7-activated HLEC and hCMEC/D3 to RBC-activated HLEC and hCMEC/D3 was calculated, using a positivity cut-off of 3, as recommended [6, 14].

### Statistical analysis

Categorical data were analyzed with Fisher’s test. McNemar’s test was used to compare paired proportions. The non parametric Mann-Whitney U test was used for non-normally distributed quantitative data. Significance was set at *p* < 0.05.

## Results

*P. falciparum* isolates were collected from 25 patients with uncomplicated malaria and 2 patients with severe malaria (1 with convulsions, 1 with prostration). Median age was 48 months (range 8 mouths to 20 years), and mean age was 62.0 ± 55.8 months. Parasite load ranged from 5316 to 315,000 parasites per microliter. All 27 isolates were matured in vitro and enriched by gelatin flotation. pRBC specimens were almost exclusively at the schizont stage.

### Cytoadherence

We first tested the binding of the 27 isolates to human ECs. Each preparation contained approximately 4 × 10^6^ pRBC. The number of adherent pRBC was determined microscopically. Cytoadherence values ranged from 1 to 2340 pRBC per 700 HLEC and 0 to 5040 per 700 hCMEC/D3 (Table 1). The number of adherent uninfected red blood cells (RBC) was negligible between 0 to 10 RBC per 700 cell with mean values of 4 per 700 cells lines (Table 1). Based on their binding to ECs, the isolates were categorized as cytoadherence-positive (number of adherent pRBC >35 per 700 cells) or cytoadherence-negative (< 35 per 700 cells). The cytoadherence-positive isolates were also categorized into those with high-level cytoadherence (HLC: > 1000 adherent pRBC per 700 cells), moderate cytoadherence (MLC: ≥100 pRBC per 700 cells) and low-level cytoadherence (LLC: < 100 per 700 cells).

**Table 1:** Cytoadherence and apoptosis of HLEC and hCMEC/D3 mediated by *P. falciparum* infected red blood cell

Pf isolate	Clinical status	Number of pRBC adhered per 700 cells		Mean OD direct contact mediated apoptosis (SD)		Mean OD soluble factor mediated apoptosis (SD)	
		hCMEC/D3	HLEC	HLEC	hCMEC/D3	HLEC	hCMEC/ D3
F1	UN	1	641	0.99 (0.05)	9.08 (0.05)	2.23 (0.09)	8.04 (0.61)
F2	UN	1	1	1.57 (0.12)	8.73 (0.62)	0.83 (0.04)	5.41 (0.07)
F3	UN	25	41	0.86 (0.04)	1.12 (0.09)	0.71 (0/09)	0.46 (0.05)
3D7^1^	-	254	415	3.15	3.79	1.25	4.52
RBC^1^	-	1	0	0.39	0.49	0.25	0.41
F4	UN	3152	201	2.79 (0.07)	1.68 (0.14)	0.74 (0.04)	0.93 (0.05)
F5	UN	806	54	1.79 (0.08)	1.57 (0.11)	0.62 (0.02)	0.63 (0.18)
3D7^2^	-	279	361	3.65	3.21	1.05	4.02
RBC^2^	-	3	8	0.67	0.23	0.42	0.29
F15	UN	69	81	6.39 (0.46)	11.37 (0.21)	2.23 (0.07)	8.59 (0.23)
F16	UN	3	9	1.00 (0.04)	0.68 (0.09)	0.41 (0.05)	1.70 (0.11)
F17	UN	9	41	0.82 (0.09)	0.84 (0.04)	0.73 (0.04)	8.73 (0.69)
3D7^3^	-	291	541	3.19	3.52	1.88	4.45
RBC^3^	-	0	0	0.95	0.32	0.89	0.48
F19	UN	7	11	0.71 (0.03)	0.69 (0.06)	0.60 (0.05)	9.08 (0.09)
3D7^4^	-	213	245	3.43	3.91	1.25	4.58
RBC^4^	-	10	6	0.20	0.88	0.11	0.61
F20	UN	51	157	2.55 (0.08)	0.92 (0.13)	0.63 (0.04)	0.54 (0.06)
F21	UN	0	1	2.07 (0.09)	0.95 (0.06)	1.56 (0.30)	0.59 (0.08)
F23	UN	5	54	2.87 (0.09)	0.89 (0.01)	1.09 (0.06)	0.74 (0.03)
3D7^5^	-	259	354	3.00	3.25	1.56	4.12
RBC^5^	-	2	2	0.38	0.14	0.46	0.23
F25^a^	SM	5040	2340	0.40 (0.12)	0.74 (0.04)	1.20 (0.10)	0.55 (0.04)
F26^b^	SM	1	3	2.08 (0.19)	3.09 (0.06)	3.48 (0.03)	1.88 (0.15)
F27	UN	1652	224	0.63 (0.04)	0.32 (0.11)	1.09 (0.11)	0.66 (0.04)
F28	UN	3	5	2.07 (0.09)	0.42 (0.04)	3.53 (0.08)	0.88 (0.16)
3D7^6^	-	368	463	3.65	4.01	1.12	4.85
RBC^6^	-	3	2	0.52	0.26	0.44	0.31
F29	UN	231	87	2.07 (0.10)	1.62 (0.06)	3.40 (0.10)	3.81 (0.11)
F30	UN	473	286	4.12 (0.14)	5.43 (0.26)	1.97 (0.04)	8.68 (0.13)
F31	UN	337	307	0.38 (0.12)	1.30 (0.34)	4.87 (0.10)	6.75 (0.18)
F33	UN	7	31	1.46 (0.09)	4.74 (0.20)	2.49 (0.06)	1.74 (0.05)
3D7^7^	-	363	435	3.02	3.85	1.00	5.24
RBC^7^	-	0	1	0.54	0.45	0.35	0.15
F34	UN	10	67	1.10 (0.07)	2.23 (0.14)	1.84 (0.08)	2.73 (0.05)
F32	UN	70	157	1.05 (0.07)	4.32 (0.17)	1.07 (0.05)	1.91 (0.11)
3D7^8^	-	440	522	3.75	4.12	1.89	5.87
RBC^8^	-	1	1	0.23	0.46	0.23	0.31
F35	UN	180	101	1.78 (0.11)	1.44 (0.10)	1.76 (0.14)	4.47 (0.09)
F36	UN	7	11	0.94 (0.07)	1.67 (0.08)	0.99 (0.01)	5.92 (0.09)
F37	UN	3	2	3.39 (0.30)	3.00 (0.15)	2.23 (0.04)	6.72 (0.10)
3D7^9^	-	304	354	3.15	3.82	1.46	4.54
RBC^9^	-	3	0	0.65	0.24	0.69	0.36
F39	UN	1	21	1.57 (0.12)	1.53 (0.07)	0.44 (0.06)	1.03 (0.05)
F40	UN	70	196	3.79 (0.14)	3.50 (0.05)	0.50 (0.04)	0.99 (0.01)
3D7^10^	-	343	398	3.05	3.81	0.96	4.21
RBC^10^	-	-	-	0.52	0.26	0.66	0.17

*Pf P. falciparum*, *UN* uncomplicated malaria, *SM* severe malaria. The number of adherent pRBC was determined microscopically; *OD* optical density. The mean OD was determined for each isolate. Using a positivity cut-off of 3; 3D7^1^: positive control for the first experiment. 3D7^2.3.…10^: positive control for the remaining experiments; RBC^1^: negative control for the first experiment. RBC^2.3.…10^: negative control for the remaining experiments

^a^Patient with convulsions

^b^Patient with prostration

#### HLEC

Seventeen (63.0%) of the 27 isolates bound to HLEC. Only one isolate (F25) exhibited high-level adherence “Table 1”.

#### hCMEC/D3

Twelve (44.4%) of the 27 isolates adhered to hCMEC/D3. Of these 12 isolates, 3 (F4, F25 and F27) exhibited high-level binding (Table 1).

#### **HLEC** and **hCMEC/D3**

Twelve isolates (44.4%) cytoadhered to both lung and cerebral ECs (Table 1). The mean number of adherent pRBCs was 157 ± 454 and 422 ± 1143 per 700 cells for HLEC and hCMEC/D3, respectively (*p* > 0.05).

### Apoptosis

As EC apoptosis is independent of pRBC cytoadherence (binding of parasitized red blood cells to endothelial cells via parasite ligands and endothelial cell receptors) and requires only direct cell-cell contact (direct contact cell to cell without any cytoadherence), we analyzed the capacity of the 27 isolates to induce HLEC and hCMEC/D3 apoptosis in both contact and non contact experiments. A total of 16 isolates (59.3%) were able to induce EC apoptosis, either by contact (with or without cytoadherence) or by the release of soluble factors (*p* > 0.05) “Table 1”.

#### Contact coculture

Nine (9) isolates (33.3%) induced apoptosis by contact. These represented 56.3% of the 16 apoptogenic isolates. All 9 isolates induced hCMEC/D3 apoptosis (mean OD = 5.9 ± 3.0), but only 4 induced HLEC apoptosis (mean OD = 4.4 ± 1.4) (Table 1). Among the 9 isolates that were apoptogenic by contact, 4 isolates cytoadhered (Table 1). EC apoptosis induced by 5 other isolates also occurred on contact, but did not cytoadhere (OD range 3 to 8.7).

#### Non-contact coculture

We then determined whether pRBC could induce EC apoptosis via the release of diffusible soluble stimuli in non contact experiments. The pRBC specimens were co-incubated with ECs in Transwel plates. Thirteen (13) (48.1%) of the 27 isolates were able to kill ECs via diffusible factors, representing 81.3% of the 16 apoptogenic isolates. Eleven (11) of the 16 apoptogenic isolates were able to kill hCMEC/D3, whereas only four isolates were able to kill HLEC. Two isolates (F29 and F31) were able to kill both lung and cerebral ECs. Seven apoptogenic isolates killed ECs by only one mechanism (soluble factors).

### Endothelial cell susceptibility

**Fig. 2:**
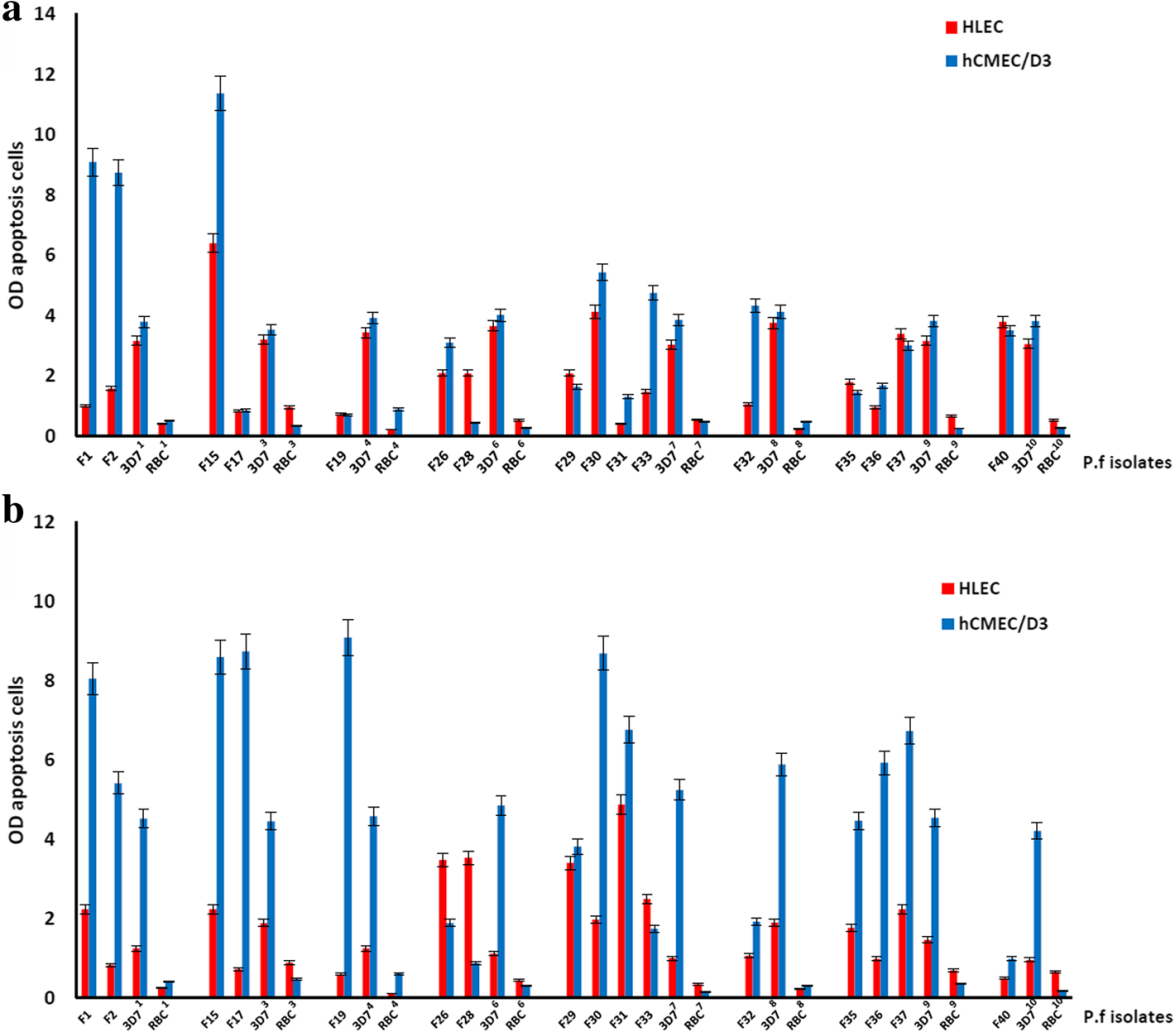
PRBC-mediated EC apoptosis. **a**) PRBC-mediated EC apoptosis by direct contact with and without cytoadherence The ability of field isolates to induce EC apoptosis was the same for hCMEC/D3 (9 apoptogenic PRBCs) and HLEC (4 apoptogenic PRBCs) (*p* > 0.05) and the mean optical density was also similar (*p* > 0.05); **b**) PRBC-mediated EC apoptosis by release of soluble factors. hCMEC/D3 were more susceptible to apoptosis mediated by soluble factors (11 apoptogenic PRBCs) than were HLEC (4 apoptogenic PRBCs) (*p* < 0.05) but intensity (mean OD ratio) of this apoptosis was higher with hCMEC/D3 (*p* < 0.01). *P. falciparum* field isolates trigger cerebral cells apoptosis than lung cells (*p* < 0.01)

Seven of the apoptogenic isolates induced both cerebral and pulmonary EC apoptosis, 8 induced only hCMEC/D3 apoptosis, and 1 induced only HLEC apoptosis. Finally, 15 of the apoptogenic isolates induced hCMEC/D3 apoptosis, and 8 HLEC apoptosis (Fig. 2). Also, the mean OD of these 15 apoptogenic isolates targeting hCMEC/D3 is higher than that of the 8 apoptogenic isolates targeting HLEC apoptosis (6.25 ± 2.22 vs 4.12 ± 1.04 respectively; *p* < 0.01). Thus, cerebral ECs were more susceptible than lung ECs to pRBC-induced apoptosis.

Half of the apoptogenic isolates triggered only hCMEC/D3 cell death (mean OD = 5.8 ± 2.1). Four of these isolates acted via soluble factors, one via direct contact without cytoadherence, two via both contact without cytoadherence and soluble factors, and the last by cytoadherence. One isolate specifically induced HLEC apoptosis (OD = 3.53) via diffusible soluble factors. All the isolates able to kill HLEC (OD range 3.4 to 6.4) in contact coculture were also able to kill hCMEC/D3 in contact coculture (OD range 3.0 to 11.4). HLEC apoptosis was induced by contact or by diffusible factors, but never by both. For example, F26, F28, F29 and F31 pRBC induced HLEC apoptosis via soluble factors but not by direct contact or cytoadhérence.

## Discussion

Knowledge of the nature and role of parasite ligands and host cell receptors associated with malaria severity is crucial for improving or developing therapeutic strategies that combine both parasite elimination and endothelial cell protection. Here, to limit possible artifacts linked to parasite adaptation to in vitro culture, we used field isolates with a minimum of ex vivo manipulation. Coculture of RBC infected by these isolates with hCMEC/D3 and HLEC was carried out in contact and non contact conditions to assess the capacity of RBC infected by these isolates to bind to ECs and to induce apoptosis, and also to determine the general mechanism of action (contact, cytoadherence and/or soluble factors). RBC parasitized-3D7 strain (in vitro maintained culture) and uninfected RBC were used respectively as positive and negative controls for both adherence and apoptosis assays as previously documented [6, 9, 14, 15, 21, 26]. The use of uninfected RBC as negative control instead of non-adherent *P. falciparum* lines is more linked to the scarcity of valuable non cytoadherent parasite line such as *P. falciparum* D10 described by Anders et al. in 1983 [27] and this does not have any influence on our results. pRBC-mediated HLEC apoptosis has been already investigated by means of transmission electron microscopy, annexin V assay, caspase activity assay, and nucleosome release ELISA [6, 21]. The ECs used in this study were first examined for ICAM-1 and CD36 expression. The hCMEC/D3 expressed ICAM but not CD36, while HLEC expressed both receptors.

We found that 59% of the pRBC specimens triggered EC apoptosis, confirming that pRBC clinical isolates can induce lung [9, 15, 21] and brain EC apoptosis [21, 28]. However, in previous studies only about 20% of clinical isolates induced human lung EC apoptosis, compared to 59% of our isolates [9, 15].

The main finding of this study is that *P. falciparum* induced preferential EC apoptosis: half of the 16 apoptogenic isolates specifically targeted hCMEC/D3 cells, while only one specifically targeted HLEC, and seven targeted both cell types. Whether these cell types differ in their intrinsic susceptibility to apoptogenic stimuli was not assessed and so it is uncertain whether these findings can be generalized to the situation in vivo. However, if a similar difference in susceptibility to *P. falciparum*-induced apoptosis occurs in vivo, this might be important in the pathogenesis of severe malaria. Overall, 15 (94%) of the 16 apoptogenic isolates triggered brain EC apoptosis, but only 8 (50%) killed HLEC (*p* < 0.05). Three pRBC phenotypes were observed: the first exclusively killed hCMEC/D3, the second only killed HLEC, and the last killed both ECs (mixed phenotype). Evidence of EC activation and apoptosis leading to altered vascular integrity and blood pulmonary/brain barrier breakdown, has been found during severe malaria [6, 8, 12, 29]. We suspected that patients with severe malaria might be infected with strains preferentially inducing either brain or lung EC apoptosis, but almost all our patients had uncomplicated malaria, meaning we were unable to test this hypothesis.

In contact experiments, 9 isolates induced both HLEC and hCMEC/D3 apoptosis. Of these, 4 isolates acted via cytoadherence. All 4 of these latter isolates (F15, F30, F32 and F40) were able to bind to hCMEC/D3 and HLEC. This is in line with two studies using field isolates in which as many as half of the pRBC samples that triggered EC apoptosis acted through cytoadherence [9, 15].

The transduction signal triggered by pRBC adherence is unclear, but cross-linking of pRBC adhesins on the EC surface is known to induce apoptosis [30]. pRBC binding specifically to CD36 induces EC apoptosis mediated by p59/fyn as well as by caspases [31]. Working with *P. falciparum* field isolates from Gabon, we have previously demonstrated that the ability of pRBC to trigger human lung EC apoptosis is linked to the expression of genes encoding PALPF [14]. We also demonstrated that selected PALPF transcripts, such as PFD0875c and MAL13P1.206, are involved in parasite cytoadherence [14]. As some isolates can induce hCMEC/D3 apoptosis by cytoadherence, these PALPFs might be involved in hCMEC/D3 apoptosis. This hypothesis is supported by previous work showing that, in brain EC coculture, some PALPF transcripts, particularly PALPF-5 and PALF-2, are up regulated in apoptogenic strains by comparison with non apoptogenic strains [21]. Binding of these PALPFs or other parasite ligands to EC-CD36 and EC-ICAM-1 may activate the Rho kinase signaling pathway that can trigger cell apoptosis. Indeed, CD36 and ICAM-1 are the receptors most commonly used by clinical isolates to survive, even though pRBC adhesion involves many other receptors [32–34].

Our results conflict with those of N’Dilimabaka et al. who found that pRBC binding was not required for EC apoptosis. These authors used *P. falciparum* field isolates adapted in vitro, whereas we used pRBC freshly collected from malaria patients. Field isolates maintained in vitro undergo clonal phenotypic variations every 48 h, corresponding to intra-erythrocytic development. This phenotypic variation, together with stress generated by in vitro conditions, may influence parasite gene expression and other characteristics.

Among the nine isolates that were apoptogenic in contact experiments, five (F1, F2, F26, F33 and F37) acted by physical contact. Four isolates (F1, F2, F26 and F33) specifically killed hCMEC/D3 while isolate F37 killed both EC types. None of these five last isolates cytoadhere to the EC they killed. This confirms that direct contact between pRBC and EC, without cytoadherence, is sufficient to trigger EC apoptosis [21]. Five of the 16 apoptogenic isolates in our study required direct contact to induce EC apoptosis. This physical contact might induce over-expression of PALPF genes different from those associated with cytoadherence. PALPFs consist mainly of trans-membrane proteins, few of which are involved in cytoadherence [14, 15].

Two-thirds of the apoptogenic pRBC samples killed hCMEC/D3 via soluble factors, compared to only one-quarter for HLEC. Similar results have been obtained with HLEC by Zang Edou et al. 2010. Isolates F29 and F31 were able to kill both ECs. Interestingly, seven apoptogenic isolates killed hCMEC/D3 or HLEC exclusively via soluble factors, and were unable to induce EC apoptosis when placed in direct contact. It appears that both mechanisms (cytoadherence and direct contact) are inhibited. Our results suggest that coculture contact inhibits the expression of genes encoding PALPF soluble antigens in these pRBCs. By contrast, two-thirds of the pRBCs inducing apoptosis by cytoadherence or direct contact were also able to trigger apoptosis via soluble factors. Perhaps all the genes coding for PALPF proteins (adhesins and soluble antigens) were expressed in these latter isolates conferring therefore the capacity of using more than one stimulus.

Finally, we suggest that the three stimuli may trigger the same signaling pathway. PALPF adhesins and soluble antigens expressed by pRBC are able to trigger EC apoptosis via Rho kinase activation. However, the mechanisms by which some pRBCs preferentially target brain versus lung ECs are unknown. The cerebral associated isolate (CAI) (a phenotype killing only hCMEC/D3) express PALPF antigens, the nature and expression kinetics of which are different to those of PALPF expressed by pulmonary associated isolate (PAI) (a phenotype specifically killing HLEC). In this case, the nature and/or affinity of the EC receptors recognized by these PALPF ligands would be also different.

## Conclusion

*P. falciparum* uses at least one of the three stimuli (cytoadherence, direct contact and soluble factors) to induce EC apoptosis. The study indicates that brain EC was more susceptible to apoptosis triggered by *P. falciparum* compared to the lung EC and may provide new insights into host-parasite interactions.

## Abbreviations

BBB: Blood-Brain-Barrier
CCm: Cerebral Culture Medium
EC: Endothelial Cell
hCMEC/D3: Human Cerebral Microvascular Endothelial Cells-D3
HLEC: Human Lung Endothelial Cell
PALPFs: *Plasmodium* Apoptosis-Linked Pathogenicity Factors
PCm: Pulmonary Culture Medium
pRBC: Parasitized Red Blood Cell
RBC: Red Blood Cells
